# High education accelerates cognitive decline in dementia: A brief report from the population-based NEDICES cohort

**DOI:** 10.1590/1980-57642016dn11-030012

**Published:** 2017

**Authors:** Israel Contador, Félix Bermejo-Pareja, D. Lora Pablos, Alberto Villarejo, Julián Benito-León

**Affiliations:** 1 Department of Basic Psychology, Psychobiology and Methodology of Behavioral Science. University of Salamanca, Salamanca, Spain.; 2 Clinical Research Unit (Imas12), University Hospital "12 de Octubre", Madrid, Spain.; 3 The Biomedical Research Centre Network for Neurodegenerative Diseases (CIBERNED), Carlos III Research Institute, Madrid, Spain.; 4 Faculty of Medicine, Complutense University, Madrid, Spain.; 5 Research Institute of Hospital "12 de Octubre" (i+12), Epidemiology Section, Madrid, Spain.; 6 Department of Neurology. University Hospital "12 de Octubre", Madrid, Spain.

**Keywords:** dementia, education, cognitive decline, population-based, demência, educação, declínio cognitivo, base populacional

## Abstract

High education has been associated with faster cognitive decline after diagnosis of Alzheimer's disease (AD), but it is unclear whether these findings extend to other dementia subtypes.

**OBJECTIVE:**

We investigated whether educational attainment influences the cognitive trajectories of older adults with different dementia subtypes.

**METHODS:**

All participants were selected from NEDICES, a prospective population-based cohort study of Spanish older adults. A total sample of 53 individuals with dementia completed the MMSE-37 at Times 1 and 2 (mean follow-up=2.8±0.5 years) to assess cognitive decline.

**RESULTS:**

At follow-up, MMSE-37 scores had decreased by 3.34±4.98 points in low-educated individuals with dementia versus 7.90±4.88 points in high-educated subjects (effect size (r)=0.32, p=0.02).

**CONCLUSION:**

Educational level influenced the cognitive trajectories of patients with dementia assessed by the MMSE-37.

## RESUMO

A educação mais alta tem sido associada com um declínio cognitive mais rápido após o diagnóstico de doença de Alzheimer (DA), mas não está claro se estes achados podem ser extendidos a outros subtipos de demência.

**OBJETIVO:**

Nós investigamos se o nível educacional alcançado influencia as trajetórias cognitivas de adultos idosos com diferentes subtipos de demência.

**MÉTODOS:**

Todos os participantes foram selecionados do NEDICES, um estudo de coorte prospectivo de base populacional de idosos adultos espanhóis. Uma amostra total de 53 indivíduos com demência completaram o MEEM-37 nos momentos 1 e 2 (acompanhamento médio de 2.8± 0.5 anos) para avaliação do declínio cognitive.

**RESULTADOS:**

No seguimento, o MEEM-37 declinou 3.34±4.98 pontos em indivíduos de baixa escolaridade com demência *versus* 7.90±4.88 pontos entre os altamente escolarizados (tamanho do efeito (r)=0.32, p=0.02).

**CONCLUSÃO:**

O nível educacional influenciou as trajetórias cognitivas de pacientes com demência avaliados pelo MEEM-37.

## INTRODUCTION

It is known that lower education is associated with an increased risk of dementia.^1^ However, high-educated individuals with dementia have a poorer prognosis (e.g., mortality) due to the accumulation of more pathological changes after diagnosis.^2^ In this respect, a recent review has indicated that high education leads to more rapid cognitive decline,^3^ but conflicting results have emerged from mixed samples (i.e., different dementia subtypes) using global cognitive measures.^4^^,^ 5 The present study explored the effect of educational attainment on cognitive decline in individuals with dementia from a population-based longitudinal cohort.

## METHODS

### Participants.

This analysis is drawn from the "Neurological Disorders in Central Spain" (NEDICES), a population-based survey of the main age-related health problems of older Spaniards (65 years or older) in central Spain (Lista, Arévalo and Margaritas). Detailed information about the study (assessments, waves) is available elsewhere.^6^^,^ 7 Each participant or their caregivers (in the case of individuals with severe cognitive impairment) signed a written informed consent form, and two ethical standards committees on human research (University Hospitals "12 de Octubre" and "La Princesa", Madrid) approved the protocol of the study.

### Measures.

The extended 37-item Spanish version of the Mini-Mental State Examination (MMSE-37) was used as the outcome measure to assess global cognitive decline from the baseline survey (1994-1995) to the follow-up (1997-1998). Levels of educational attainment were registered as follows: illiteracy, can read and write, primary education, and secondary and higher education. Finally, the adapted Charlson index was used to assess participants' comorbidity.^8^

### Procedure.

The NEDICES study was carried out in two phases: door-to-door screening of eligible people (Phase 1) and neurological examination of those individuals who screened positive (Phase 2). A total of 5278 older citizens were assessed at baseline (1994–1995) using a 500-item screening questionnaire to collect data on demographics, medical conditions and current medication. In addition, participants underwent the World Health Organization (WHO) screening protocol for dementia, which included the MMSE-37 and the 11-item Spanish version of Pfeffer's Functional Activities Questionnaire.^9^^,^ 10 This (in person) assessment was done by trained interviewers in two cross-sectional surveys: baseline wave (1994-5) and incidence wave (1997-8).

Each person underwent initial screening for cognitive impairment and if testing positive, a neurological examination was performed at the National Health Service clinics or at home. The initial screen was considered positive if: [1] the individual scored <24 points on the 37-item version of the MMSE and >5 points on the Pfeffer questionnaire; or [2] the individual could not provide an answer on the 37-item version of the MMSE or on the Pfeffer questionnaire (direct screening); or [3] the individual or proxy gave information pointing to suspected cognitive decline. The diagnosis of dementia was subsequently made by the consensus of two neurologists according to the Diagnostic and Statistical Manual of Mental Disorders (DSM–IV). Alzheimer-type dementia (AD) was diagnosed according to NINCDS-ADRDA criteria, whereas for vascular dementia (VaD), the DSM-IV was employed. Other categories, such as dementia associated with Parkinson's disease, dementia with Lewy bodies, or longstanding Parkinsonism (more than six months) and secondary dementia (known or probable cause of specific dementia), were also established.^7^ The neurological examination comprised a clinical history concerning cognitive decline, a general neurological examination and a mental status interview.

### Statistical analysis.

In this study, education level was dichotomized into high (1=individuals with a primary school certificate or higher) and low (0=illiterate subjects and individuals who can only read and write) for the analysis. The groups (high vs. low educational attainment) were compared using the Chi-square test (nominal variables) and Student's t-test for independent samples (quantitative variables). The change in MMSE-37 score was calculated as follows: baseline score (1994-1995=Time 1) – follow-up score (1997-1998=Time 2). Due to the absence of normality of the MMSE-37 scores, the significance of the change was analyzed using nonparametric comparisons (Mann-Whitney test). Specific effect sizes (r) for non-normal distributions were computed as r=z/√n, where z is the standardized statistic (with normal distribution) and n is the sample size.^11^ Analyses were performed with SPSS (IBM SPSS Statistics version 21).

## RESULTS

Of the 306 participants with a diagnosis of dementia at baseline (1994-1995), 128 did not complete the MMSE-37 because they refused or were unavailable for face-to-face evaluation. These subjects were excluded from the statistical analyses. Of the 178 remaining individuals, 53 (35 with Alzheimer's disease, 10 VaD and 8 other types) completed the MMSE-37 assessments at Time 2 (mean follow-up=2.8±0.5 years). Individuals with dementia who did not complete the MMSE-37 in both waves were older (83.1 vs. 80.2, t=2.67, p<0.01), higher educated (83 with secondary school or higher (33.9%) vs. 10 (18.9%), χ^2^=4.57, p<0.05) and showed greater cognitive impairment at baseline (14.78±6.82 vs. 16.85±6.67, t= –1.88, p=0.05) versus participants who completed the two assessments.

Table 1 shows the descriptive statistics of the individuals with dementia stratified by educational level. No significant differences in any characteristics emerged between high and low education groups at baseline.

**Table 1:** Descriptive characteristics of the population with dementia.

Dementia group (N=53)					
Variable		Low education (N=43)	High education (N =10)	t/c2	p
Age		80.0	81.2 (6.9)	–0.55	0.58
Sex (% female)		67.4	70	0.02	0.87
Educational attainment	Illiterates/Read & Write	21/22	–		
	Primary school/Secondary and higher	–	8/2		
Dementia type (AD/O)		28/15	7/3	2.69	0.44
Severity of dementia↑ (mild/moderate/severe)		24/18/1	7/3/0	1.41	0.92
Charlson Index		4.1 (1.4)	3.9 (1.1)	0.54	0.58
MMSE (Time 1)		16.2 (6.1)	19.3 (7.6)	–1.33	0.18
Pfeffer (Baseline)		18.3 (9.8)	16.8 (9.3)	0.44	0.65

AD: Alzheimer disease; O: Other dementia types; ↑Clinical severity of dementia was established according to DSM–III–R criteria.

**Figure 1:**
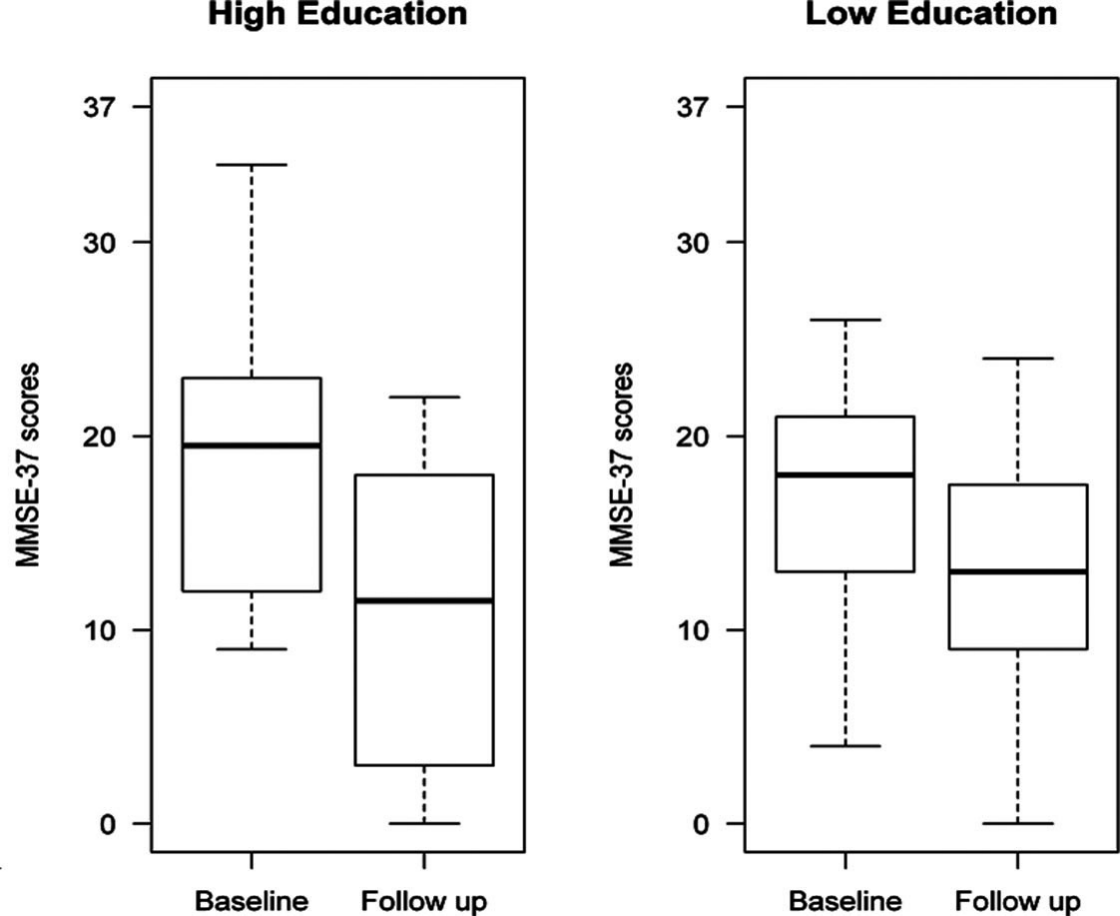
Cognitive evolution of participants with dementia according to educational level: 3-year follow-up.

At follow-up, MMSE-37 scores had decreased by 3.34±4.98 points in individuals with lower education vs. 7.90±4.88 points in patients with high educational level (effect size (r)=0.32, Mann-Whitney p=0.02). MMSE-37 scores (Times 1 and 2) of patients with dementia according to level of education are shown in Figure 1.

## DISCUSSION

This scientific report found that high-educated individuals with dementia had significantly faster cognitive decline compared to the low-educated group at the 3-year follow-up. Our findings are consistent with previous studies based on AD and mixed samples with other subtypes of dementia.^5^^,^ 12 Basically, these observations suggest a more advanced level of neuropathology in high-educated individuals with dementia at similar levels of dementia severity. However, Aguero-Torres et al.^4^ failed to confirm this effect using an adjusted multiple regression model with only 74 individuals who completed the follow-up.

This study has several limitations. First, cognitive decline was assessed by the MMSE-37, a global measure of decline which limits generalization of findings to specific cognitive domains. Nevertheless, comparable results have been obtained using memory performance.^13^ Second, a percentage of demented subjects could not be reached at follow-up, and sample size was reduced. Essentially, non-selected individuals were older, more highly educated and more cognitively impaired than the selected sample. All these factors have been linked to an increased risk of mortality, supporting an explanation of attrition in population-based studies.^1^ In any case, the influence of education was significant (i.e., small-medium effect size) in two population-based groups (high vs. low education) well-matched for other sociodemographic and clinical characteristics.

To sum up, high-educated individuals with dementia showed faster cognitive progression at the 3-year follow-up. This research encourages us to investigate the role of education and other cognitive reserve proxies in cognitive changes related to other neurological conditions. Thus, education-corrected norms are needed to evaluate cognitive performance in people with Parkinson's disease or essential tremor, but its influence on cognitive changes over time has been little investigated in these conditions.^14^ The ultimate aim of this approach would be to verify whether modulating factors of cognitive trajectories in people with AD can be applied to individuals with PD or other neurological conditions.
